# Hepatitis C virus NS4B carboxy terminal domain is a membrane binding domain

**DOI:** 10.1186/1743-422X-6-62

**Published:** 2009-05-25

**Authors:** Jolanda MP Liefhebber, Bernd W Brandt, Rene Broer, Willy JM Spaan, Hans C van Leeuwen

**Affiliations:** 1 Department of Medical Microbiology, Center of Infectious Diseases, Leiden University Medical Center, 2300 RC Leiden, the Netherlands; 2 Centre for Integrative Bioinformatics (IBIVU), VU University Amsterdam, the Netherlands

## Abstract

**Background:**

Hepatitis C virus (HCV) induces membrane rearrangements during replication. All HCV proteins are associated to membranes, pointing out the importance of membranes for HCV. Non structural protein 4B (NS4B) has been reported to induce cellular membrane alterations like the membranous web. Four transmembrane segments in the middle of the protein anchor NS4B to membranes. An amphipatic helix at the amino-terminus attaches to membranes as well. The carboxy-terminal domain (CTD) of NS4B is highly conserved in Hepaciviruses, though its function remains unknown.

**Results:**

A cytosolic localization is predicted for the NS4B-CTD. However, using membrane floatation assays and immunofluorescence, we now show targeting of the NS4B-CTD to membranes. Furthermore, a profile-profile search, with an HCV NS4B-CTD multiple sequence alignment, indicates sequence similarity to the membrane binding domain of prokaryotic D-lactate dehydrogenase (d-LDH). The crystal structure of E. coli d-LDH suggests that the region similar to NS4B-CTD is located in the membrane binding domain (MBD) of d-LDH, implying analogy in membrane association. Targeting of d-LDH to membranes occurs via electrostatic interactions of positive residues on the outside of the protein with negative head groups of lipids. To verify that anchorage of d-LDH MBD and NS4B-CTD is analogous, NS4B-CTD mutants were designed to disrupt these electrostatic interactions. Membrane association was confirmed by swopping the membrane contacting helix of d-LDH with the corresponding domain of the 4B-CTD. Furthermore, the functionality of these residues was tested in the HCV replicon system.

**Conclusion:**

Together these data show that NS4B-CTD is associated to membranes, similar to the prokaryotic d-LDH MBD, and is important for replication.

## Background

Hepatitis C virus (HCV) preferentially infects hepatocytes [1]. Although this does not have a direct cytopathic effect, infection often becomes persistent, slowly progressing into chronic liver diseases like cirrhosis and hepatocellular carcinoma [2,3]. Phylogeny of HCV places this positive sensed RNA virus, within the genus *Hepaciviruses *of the family *Flaviviridae *[4]. The single stranded RNA genome contains one open reading frame flanked by two non-translational regions (NTRs) at the 5' and 3'-end. An internal ribosomal entry site in the 5'-NTR facilitates the translation of the polyprotein [5]. Cellular and viral-encoded proteases process the polyprotein into three structural proteins (core and two glycoproteins, E1 and E2), a hydrophobic peptide p7 and six non-structural (NS) proteins [6,7].

During infection the conformation of cellular host membranes changes in a number of ways. One of these membrane alterations is the membranous web (MW), composed of small vesicles embedded in a membrane matrix [8]. Ultrastructural analysis of HCV replicon cells in combination with labeling of viral RNA revealed that this membranous web is the site of RNA synthesis [8].

The non-structural (NS) proteins NS3 to NS5B are required for viral replication [9]. They localize to the cytosolic leaflet of membranes derived from the endoplasmic reticulum (ER) [10]. NS3 possesses RNA helicase as well as protease activity. Membrane anchoring of NS3 is mediated through an amphipatic helix at the N-terminus of NS3 and a transmembrane segment in NS4A, which is also a co-factor for NS3 protease [11,12]. New HCV RNA strands are synthesised by NS5B, the RNA-dependent RNA polymerase. NS5B is targeted post-translationally to membranes via a carboxy terminal hydrophobic domain [13,14]. NS5A, a peripheral membrane binding protein, associates with lipids via an amphipatic helix at its amino-terminus [15]. Importance for both replication and virus production has been suggested for NS5A [16,17]. A central role for the integral membrane protein, NS4B, in the formation of the membranous web was suggested when Egger et al. showed that very similar structures could be induced by the NS4B protein in the absence of any other HCV proteins [18]. These NS4B induced structures were defined as swollen, partially vesiculated membranes and clustered aggregated membranes [19].

NS4B is a hydrophobic protein with a molecular weight of approximately 27 kDa and has a modular domain organization with the amino- (N) and carboxy- (C) terminal ends being cytoplasmic and a central region which is inserted in the ER membrane. A topology study of NS4B indicated that the central domain has four transmembrane segments [20,21]. The N-terminal part, approximately 70 to 90 amino acids long, has several reported functional properties. The extreme N-terminal segment of NS4B revealed the presence of a putative amphipatic helix (AH, aa 6 – 29), which mediates membrane association through its hydrophobic side [22]. Disruption of this helix alters its ability to rearrange intracellular membranes and the localization of HCV replication proteins [21,22]. The region next to this amphipatic helix is predicted to form a large amphipatic helix (aa 22 – 49), with the characteristics of a basic leucine zipper motif (bZIP) [23]. The first 72 amino acids from the N-terminus of NS4B have been suggested to be involved in multimerisation [24], which may involve intramolecular leucine zipper interactions. A post-translational relocation of the N-terminus to the ER lumen was proposed for a fraction of the NS4B pool, giving the protein a dual transmembrane topology with either four or an extra fifth transmembrane domain (TMx) [20,21]. The C-terminal domain (CTD) of NS4B is oriented towards the cytosol and seems well conserved throughout hepaciviruses. Despite this sequence conservation not much is known about the CTD, though lately several studies describe possible characteristics of the domain [24-27]. A genetic interaction between NS3 with the extreme C-terminus of NS4B has been postulated [27]. Besides protein-protein interactions [24,27], a protein-RNA interaction has also been suggested [25]. Furthermore the CTD of NS4B is involved in RNA synthesis and virus production [26].

The most widely suggested function for NS4B is the creation of a platform in the cell that concentrates the virus template, replication and host cell proteins, thereby increasing the efficiency of replication [18,28]. Alternatively, distortion of cellular membranes can reduce the transport of cell surface proteins in infected cells in order to escape from the host immune response [19]. Other functions attributed to NS4B are inhibition of host as well as viral protein translation [29,30] and modulation of NS5a hyper-phosphorylation [31]. Clearly, NS4B is involved in a wide range of activities, which seem to point to a role in modulating the host cell environment either for evasion of the host response or optimizing the setting for viral replication.

In this study we investigate the most conserved, though least characterized, domain of NS4B, the CTD. Expression of this domain in Huh7 cells, a human hepatoma cell line, revealed membrane targeting of the NS4B-CTD, in contrast to its predicted cytosolic localization. Based on similarity with D-lactate dehydrogenase (d-LDH) membrane binding domain and mutational studies, we suggest that the NS4B-CTD is a membrane binding domain. The importance of this membrane targeting during replication was analyzed in replicon studies. Taken together our results show that in addition to the N-terminus and the transmembrane domains, NS4B can associate with intracellular membranes via its CTD. Furthermore, mutational studies suggest that, for membrane targeting, positive residues in the NS4B-CTD interact with the negatively charged head groups of lipids.

## Results

### NS4B carboxy terminal domain localizes to internal membranes

**Figure 1:**
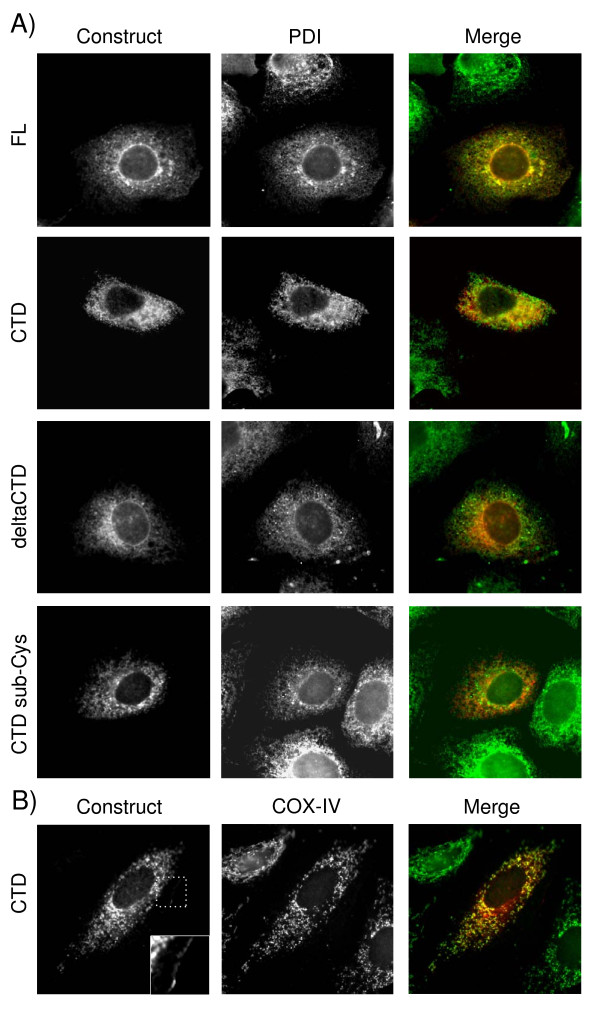
**Expression of different NS4B proteins in Huh7 cells**. Huh7 cells were transfected with NS4B full-length (FL, aa 1–261), deltaCTD (aa 1–192), CTD (aa 188–261) or CTD substitution-Cysteines (CTD sub-Cys) and 24 h later processed for indirect immunofluorescence. Cells were double labeled with antibodies reacting against Myc-epitope-tag at the C-terminal end of each protein (in red) and A. protein disulphide isomerase (PDI) or B. Cytochrome C oxidase subunit IV (COX-IV) (in green), in first and second panels respectively. Third panels show merged images.

A well-conserved part of the HCV NS4B protein is the carboxyl terminal domain (NS4B-CTD), which is also conserved within the hepacivirus genus [23]. Along with the expected cytosolic localization it proposes a separate function of the NS4B-CTD. To study the localization of NS4B-CTD various constructs were made. To each construct a Myc-epitope-tag was fused at the C-terminus as a detection epitope. These constructs were transfected into Huh7 human hepatoma cells and analyzed using immunofluorescence. Localization of the constructs was first compared to the endoplasmatic reticulum (ER), using Protein Disulphide Isomerase (PDI) as a marker. In Figure 1a, top left panel, Huh7 cells expressing full length NS4B (NS4B-FL, aa 1–261) are shown. NS4B-FL has a perinuclear and reticular staining, typical for ER. Additionally, the pattern of NS4B-FL largely overlaps with PDI. This ER-like staining confirms the previously described localization of native FL NS4B [20,32]. To our surprise, expression of the NS4B-CTD alone (aa 188–261) does not show a cytosolic staining, but displays small punctate or dot like structures throughout the cells (Fig. 1a, CTD left panel). In the overlay of NS4B-CTD and PDI some co-localization is seen between the two (Fig. 1a, CTD right panel). Together with the small punctate staining, this suggests that the NS4B-CTD might be associated to membranes.

Since the NS4B-CTD shows a dot like pattern, it might have an effect on the attachment to membranes or even localization of NS4B-FL. Therefore, an NS4B lacking the CTD (NS4B-deltaCTD, aa 1–192) was constructed and examined in immunofluorescence. As shown in Figure 1a, NS4B-deltaCTD has a perinuclear and reticular staining, like NS4B-FL and PDI, indicating an ER-like localization (Fig. 1a). Also co-transfections of NS4B-FL and NS4B-deltaCTD show similar localization (data not shown). Together this implies that the absence of CTD does not seem to alter the localization of NS4B.

Two potential lipid modification sites for palmitoylation on cysteines, suggested by Yu and colleagues [24], might render the NS4B-CTD to membranes. We therefore investigated this possibility and mutated the two cysteines (cysteines 256 and 260) of the NS4B-CTD into serines (NS4B-CTD sub-Cys) (Fig. 1a) and expressed this mutant in Huh7 cells. Localization of the NS4B-CTD sub-Cys mutant was very similar to NS4B-CTD, exhibiting small punctate structures in the cells (Fig. 1a). It shows that the dot-like membrane localization of NS4B-CTD is caused by characteristics in the domain other than the cysteines at positions 256 and 260.

### Membrane association of the carboxy terminal domain of NS4B

**Figure 2:**
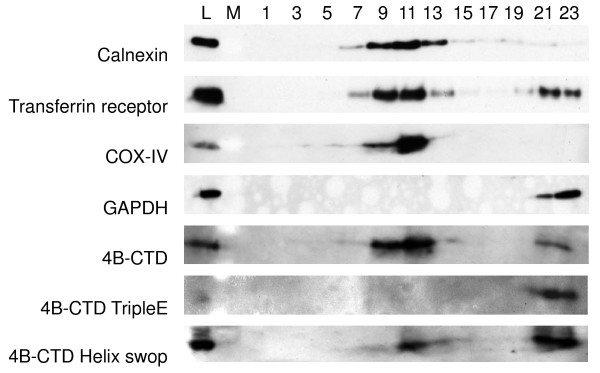
**Membrane association of NS4B carboxy terminal domain**. Huh7 cells transfected with NS4B-CTD-Myc, NS4B-CTD tripleE-Myc or NS4B-CTD Helix-swop-Myc were subjected to sucrose density gradient centrifugation. Cell lysates were loaded under a sucrose gradient from 10–80% w/v and part of the lysate was used as a loading control (L). Fractions were taken from top (fraction 1) to bottom (fraction 23) and separated by SDS-PAGE. Followed by immunoblot analysis for Calnexin, Transferrin Receptor (TfR), Cytochrome C oxidase subunit IV (COX-IV) and Glyceraldehyde 3-phosphate dehydrogenase (GAPDH). NS4B-CTD and NS4B-CTD tripleE were assayed using an antibody against Myc-epitope. M indicates where molecular weight marker was loaded.

Membrane association of proteins can be investigated in a membrane floatation assay. In such an assay, a continuous-density gradient is loaded on top of a cell extract and subjected to centrifugation. Membranes and associated proteins float into the gradient, while cytosolic proteins stay in the loaded bottom fraction. To examine the suggested membrane association characteristics of the NS4B-CTD a membrane floatation assay was performed. Figure 2 shows the results of that assay, in which a cell lysate of Huh7 cells transfected with NS4B-CTD was used. Fractions were collected from the top (10%) to the bottom (80%) of the gradient and the odd fractions were analyzed by western blotting. As a control for cytosolic proteins, glyceraldehyde 3-phosphate dehydrogenase (GAPDH) was used. As expected, GAPDH was retained in the bottom fractions 21 and 23 of the density gradient, where the cell extract was loaded (Fig. 2). Calnexin, Transferrin receptor (TfR) and Cytochrome C oxidase subunit IV (COX-IV) are transmembrane proteins and float into the gradient, they are mainly observed in fractions 9 and 11 (Fig. 2). Since calnexin, TfR and COX-IV reside on different membranes in the cell (ER, the endocytic pathway and mitochondria), their distribution differs slightly (Fig. 2). The NS4B-CTD is detected in fractions 7 to 13 and 21 and 23 with its highest signal in fraction 11 (Fig. 2). In conclusion, similar to membrane proteins the NS4B-CTD floats into the gradient, implying membrane association of the NS4B-CTD. Together, the punctate structures in immunofluorescence and the floatation into the membrane floatation gradient, suggest association of the CTD of NS4B to membranes.

### Cellular localization of the NS4B carboxy terminal domain

Since the CTD of NS4B only partially overlaps with the ER-marker, PDI (Fig. 1a), we were interested in knowing on which other membranes the NS4B-CTD resides. Therefore, co-localization studies with different organelle markers in Huh7 cells transfected with NS4B-CTD were performed. From the exocytic pathway we examined the Golgi (Giantin) and the ER-Golgi intermediate compartment (ERGIC) and found no substantial co-localization (data not shown). Similar results were obtained from co-localization studies with markers from the endocytic pathway, such as Rab5 from early endosomes, mannose-6-phosphate receptor and LAMP1, proteins that resides in late endosomes and lysosomes (data not shown). Recently, lipid droplets were demonstrated to play an important role in the HCV lifecycle [33]. However, no co-localization of lipid droplets and the NS4B-CTD was observed (data not shown). HCV proteins, Core, NS3 and NS4A are suggested to localize to or close to mitochondria [34,35]. For that reason, co-localization of mitochondria and NS4B-CTD was investigated. We could observe considerable similarity in patterns between COX-IV, a mitochondrial protein marker and the NS4B-CTD (Fig. 1b). However, the overlap is not complete. Even though we did not specifically preserve the plasma membrane during immunofluorescence, we could occasionally see a fraction of NS4B-CTD at the plasma membrane (Fig. 1b. Inset). Taken together the CTD of NS4B seems to be mainly targeted to mitochondria, ER membranes and the plasma membrane.

### Profile searches with an HCV NS4B carboxy terminal domain alignment suggest similarity to Lact-deh-memb

**Figure 3:**
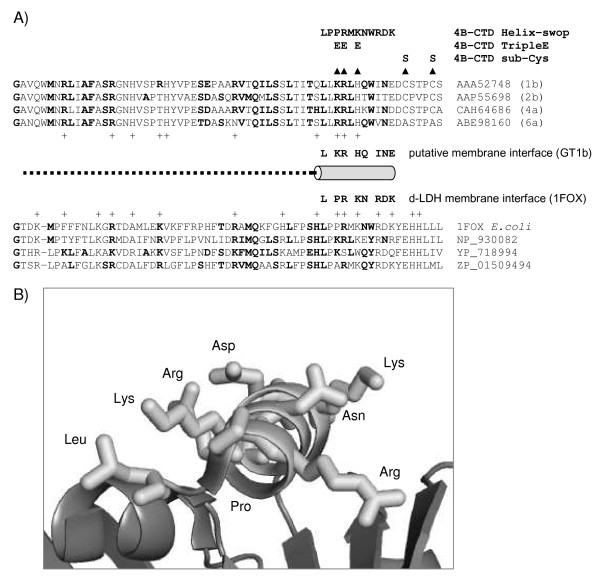
**Sequence similarity between NS4B carboxy terminal domain and the membrane binding domain of D-lactate dehydrogenase**. **A**. Multiple sequence alignment of the carboxy terminal domain of four genotypes of HCV NS4B proteins and the membrane binding domain of four d-LDH family members (referenced by their accession numbers). Bold residues highlight amino-acids present in both families. Basic residues (R, K, H) making up the potential electropositive surface are indicated (+). Dotted line indicates disordered region in the d-LDH crystal structure. Arrowheads point to mutations made in the CTD of NS4B. **B**. Ribbon representation of the membrane anchored side of d-LDH (PDB code 1F0X). Stick residues indicate the surface exposed amino-acids of the ordered membrane binding helix.

The importance of the NS4B-CTD might be reflected by the sequence conservation within hepaciviruses. Its sequence conservation may also provide a clue to its function. Identification of potentially remote protein homologues can help to predict protein properties, like folding, structure and most importantly function. Similarity between distantly related proteins can be effectively established using profile based searches of databases of proteins families. In order to elucidate a possible function of the CTD of NS4B, we generated a multiple sequence alignment (profile) of the NS4B-CTD including all genotypes of HCV, Hepatitis GB virus A, B and C (Additional file 1), which we manually refined. Programs for profile-profile comparisons have been developed and are available as web-based tools. We used three different tools for profile-profile comparison with our HCV NS4B-CTD query profile, namely PRC [36], HHpred [37] and COMPASS [38] because each is sensitive to a different set of algorithms and the combination of the three tools reinforces independently detected relationships. This allows us to construct a consensus result with hits found by all tools. Using similar search parameters (see Materials and Methods) twelve, eight and five hits were found by PRC, HHpred and COMPASS respectively. Interestingly, only one protein family Lact-deh-memb (PF09330) was found by all three methods. This was the highest scoring profile for the three methods, next to the NS4B profile (E-values 0.057, 0.086 and 0.35 for the respective searches). According to HHpred the probability (which also includes the contribution from the secondary structure score) that lact-deh-memb is significant similar to NS4-CTD is 44.8%. Members of this Lact-deh-memb family are predominantly found in prokaryotic D-lactate dehydrogenase (d-LDH), which is a peripheral membrane respiratory enzyme located on the cytosolic site of the inner membrane [39]. Comparison of the sequence similarity between HCV NS4B-CTD and d-LDH from *E. coli*, of which the crystal structure has been resolved [39], revealed that the common region lies in the membrane binding domain (MBD) of d-LDH (Fig. 3a and Additional file 1) [40]. Thus besides apparent sequence similarity, both domains seem to perform similar functions, that is, they allow for membrane association. The MBD of d-LDH was suggested to bind nonspecifically to the membrane through (the positively charged) basic residues (Lys, Arg), interacting with the negatively charged phospholipids of the membrane, rather than penetrating the lipid bilayer [39-41]. Part of the d-LDH MBD corresponding to the CTD of NS4B is disordered in the crystal structure and is thought to form a defined structure upon binding to the membrane [39]. The central alpha helix of the d-LDH MBD (Fig. 3b) corresponds to the extreme carboxy-terminal end of NS4B-CTD. The amino-acids on the membrane interface of this alpha helix and the corresponding residues of NS4B-CTD are indicated in Figure 3a.

### Membrane targeting of NS4B carboxy terminal domain and d-LDH is comparable

**Figure 4:**
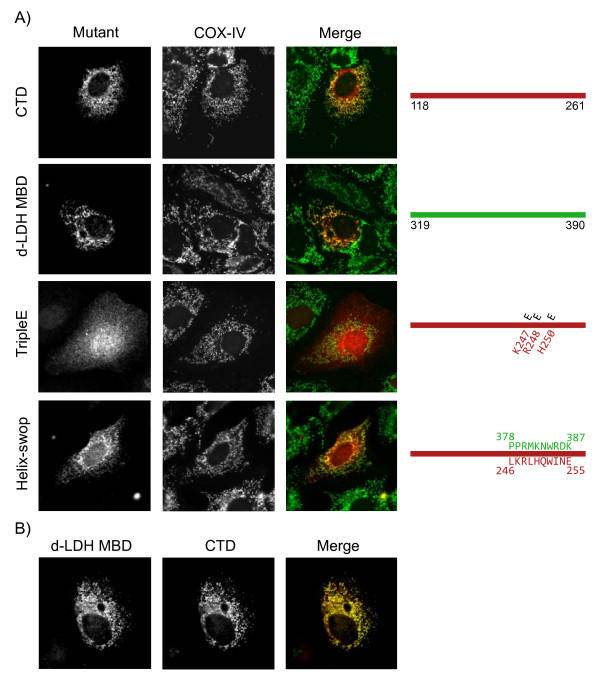
**Cellular distribution of NS4B carboxy terminal domain mutants and D-lactate dehydrogenase membrane binding domain in Huh7 cells**. **A**. The panels on the right show Huh7 cells expressing different NS4B-CTD mutants or d-LDH membrane binding domain (d-LDH MBD) after 24 h. Expression constructs are shown in red. Using COX-IV as a marker protein, mitochondria are shown in middle panels and in merged picture in green. On the right a schematic view of the different NS4B-CTD mutants is drawn; in red the sequence of NS4B-CTD-wt, in black the mutations made and in green the exchanged amino acids from the membrane contacting helix of the d-LDH-MBD. **B**. Huh7 cells were co-transfected with NS4B-CTD-HA and d-LDH MBD-Myc and analyzed by immunofluorescence after 24 h of expression. The first panel shows d-LDH MBD, which is presented as red in the merged picture. NS4B-CTD is displayed in the second panel and is shown in the merged picture as green.

Profile-profile comparison E-values in the range of 0.1–0.001 can indicate a true relationship, but require additional evidence to conclude that there is a functional parallel between NS4B-CTD and d-LDH-MBD in membrane binding [42]. Mutational studies could reveal functional similarity and were accordingly performed. D-LDH is a general membrane binding protein in *E. coli *located on the cytosolic side of the inner membrane. The position of such a protein in eukaryotic cells is unknown. Therefore, we first investigated localization of d-LDH MBD in Huh7 cells. As shown in Figure 4a d-LDH MBD (aa 319 to 390) mainly overlaps with COX-IV illustrating that when the d-LDH MBD is expressed separate from the enzyme part of the d-LDH protein, functionality of membrane binding is maintained. Moreover, co-transfection of NS4B-CTD and d-LDH MBD showed nearly complete overlap of the two patterns (Fig. 4b). Furthermore these immunofluorescence assays indicate that d-LDH MBD, a general membrane binding domain, has a preference for mitochondrial membranes in eukaryotic cells, which is comparable to the localization of NS4B-CTD (Fig. 4a), implying analogous membrane targeting of the two domains.

To test the hypothesis that the CTD of NS4B associates with the membranes in a way similar to the d-LDH MBD, we introduced mutations designed to disrupt the positive residues postulated to interact with the negative head groups of lipids [39,40] (Fig. 3a). The side chains of the d-LDH MBD pointing away from the protein, facing the membrane surface are indicated in Figure 3b. Three positively charged amino acids (Lys 247, Arg 248 and His 250) in NS4B corresponding to the structured alpha-helix in d-LDH were simultaneously replaced with a negatively charged glutamic acid (K247E/R248E/H250E; NS4B-CTD tripleE), which should not be able to bind to phospholipid heads. The NS4B-CTD tripleE mutant was expressed in Huh7 cells and membrane association was investigated using immunofluorescence and a membrane floatation assay. Mutation of all three positively charged residues results in a dramatic change of localization of the NS4B-CTD, from punctate structures in the perinuclear region to a diffuse distribution throughout the cell, possibly cytosolic (Fig. 4a, compare NS4B-CTD to NS4B-CTD tripleE). Loss of membrane association was also shown in a continuous-density gradient, in which NS4B-CTD tripleE was detected in the same fractions as the cytosolic marker GAPDH (Fig. 2).

A functional parallel can also be examined by swopping part of the membrane binding domains of two proteins. A mutant was constructed, in which we exchanged the putative membrane contacting helix of NS4B-CTD for the corresponding membrane contacting helix of the d-LDH MBD (NS4B-CTD helix-swop) (Fig. 4a). Huh7 cells expressing NS4B-CTD helix-swop display punctate structures in immunofluorescence, though the staining has a slightly more diffuse localization compared to NS4B-CTD (Fig. 4a). Similarity in patterns with COX-IV also indicated that the NS4B-CTD helix-swop is targeted to membranes while the NS4B-CTD tripleE mutant has lost membrane binding. Furthermore a membrane floatation assay showed that NS4B-CTD helix-swop is membrane associated (Fig. 2), although compared to NS4B-CTD more was observed in the non-floating fractions. Altogether these results illustrate that the CTD of NS4B can interact with membranes via the positively charged residues, comparable to d-LDH MBD.

### Positively charged residues of NS4B carboxyl terminal domain are essential for replication

**Figure 5:**
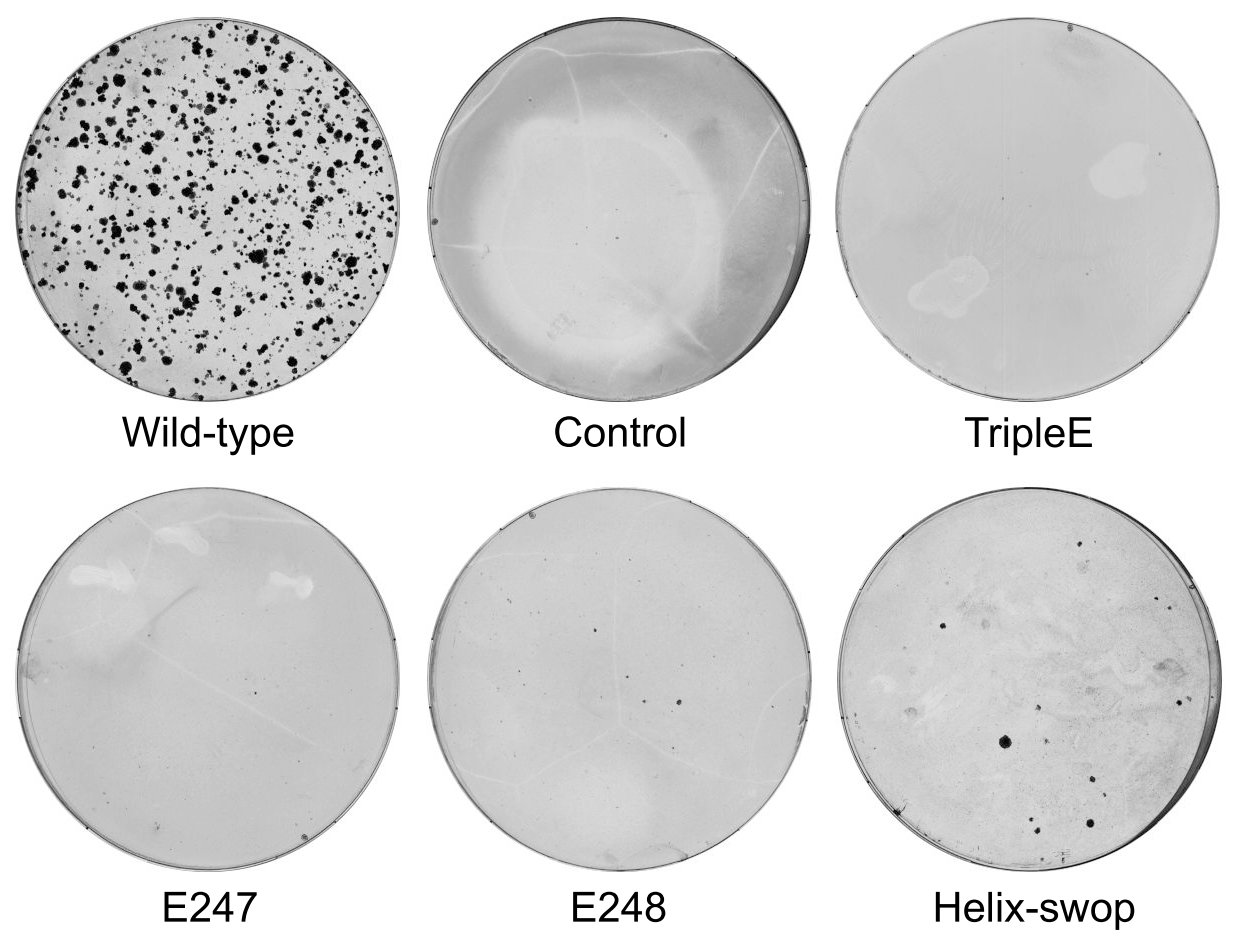
**Effect of NS4B carboxy terminal mutations on colony formation using selectable replicons**. Colony formation assay in which Huh7 cells are transfected with in vitro transcribed replicon RNA that contain NS4B-CTD mutations. Colonies were stained using Coomassie blue. Wild-type is pFK5.1. Mock transfected cells as the control. The NS4B-CTD mutations TripleE, E247, E248 and helix-swop in pFK5.1 are explained in figure 4.

To examine the importance of the NS4B-CTD positively charged residues for RNA replication, we exchanged these amino acids involved in membrane association for negatively charged glutamic acids in selectable subgenomic replicons [9]. Huh7 cells transfected with replicon RNA that carry the three negatively charged residues (NS4B-CTD tripleE) did not yield any viable colonies (Fig. 5). Moreover the single mutations K247E and R248E were replication defective and gave no colonies (Fig. 5). Thus the positive residues are clearly indispensible for viral RNA replication in cell culture, suggesting that loss in membrane association leads to a replication defect. These results, together with the possible functional parallel between d-LDH MBD and the NS4B-CTD, prompted us to swop the membrane binding helix from d-LDH MBD (PPRMKNWRDK) into replicons (helix-swop, Fig. 3a) and determine colony formation. These replicons in which eight amino acids are exchanged indeed formed several viable colonies (40 colony forming units per ug (CFU) of transfected replicon RNA) (Fig. 5). Clearly, far less colonies were formed relative to wild type (~10.000 CFU), but the replication defect from the helix swop is less than the negative charged mutations, where no colonies were formed. Two separate replicon colonies derived from the NS4B-CTD helix-swop were expanded, RNA isolated and sequenced to analyze whether they still contained the original mutations. Interestingly, the complete introduced helix was retained, confirming the importance of this membrane contacting helix.

## Discussion

Compared to other HCV proteins NS4B is the least characterized. Besides involvement in replication and induction of membrane rearrangements little is known about the function(s) of the protein. A well-conserved part of NS4B is the carboxy terminal domain (CTD) (Additional file 1), which is predicted to contain two alpha-helixes and expected to localize cytosolically [20,23]. Surprisingly, we found using different approaches that the NS4B-CTD is membrane associated. Immunofluorescence analysis of Huh7 cells expressing NS4B-CTD shows punctated structures (Fig. 1). Furthermore in a membrane floatation gradient, we could demonstrate that fractions containing floating membranes also have NS4B-CTD (Fig. 2). Using profile-profile searches, we found similarity between the CTD of NS4B and the membrane binding domain (MBD) of D-lactate dehydrogenase (d-LDH) (Fig. 3a). D-LDH is a prokaryotic respiratory enzyme that is located on the cytosolic side of the inner membrane [39]. When we expressed the MBD of d-LDH from *E. coli *in mammalian Huh7 cells and performed immunofluorescence, we observe a pattern similar to NS4B-CTD (Fig. 4a). Nearly complete overlap of both signals was shown in a co-transfection experiment of NS4B-CTD and the MBD of d-LDH (Fig. 4b), indicating a functional parallel of both domains in membrane association. D-LDH is suggested to anchor to the membrane via interactions of positively charged amino-acids with the negative heads of membrane phospholipids [39-41]. Substitution of three positive residues in the NS4B-CTD resulted in complete loss of membrane association (Fig. 4a). Together these experiments strongly suggest association of the NS4B-CTD to membranes.

The localization of NS4B-CTD to mitochondria is the most prominent (Fig. 1b and 4a). However, there is no complete co-localization as a fraction is targeted to the ER (Fig. 1a) and the plasma membrane (Fig. 1b, Inset). In addition, the d-LDH MBD, a general membrane binding domain that normally targets the enzyme towards the cytosolic side of the inner membrane of/in *E. coli *through electrostatic interactions, is largely located on mitochondrial membranes when expressed in human Huh-7 cells (Fig. 4a) [39]. The apparent preference for mitochondria might be caused by the slow turnover rate of mitochondrial membranes compared to the rapid turnover of ER and Golgi membranes [43]. A more general membrane association characteristic of the NS4B-CTD is implied by these results.

**Figure 6:**
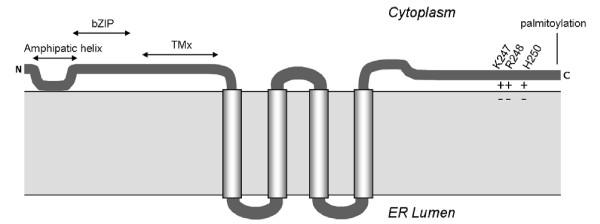
**Model of NS4B membrane association**. Schematic of the proposed topology of NS4B relative to the ER membrane and reported functional properties (see introduction). Model for NS4B-CTD membrane association is discussed in this paper. Here we propose that positive residues (amino acids are indicated) are important for membrane targeting through the interaction with the negative head groups of phospholipids. Abbreviations: Nt, Amino terminus; Ct, Carboxyl terminus; bZIP, basic leucine zipper motif; TMx, transmembrane segment X.

Given the similarity between the CTD of NS4B and the d-LDH membrane binding domain, the mode of general association to the membrane, through electrostatic interactions [39-41], might be comparable as well. When we substituted three positive residues in the NS4B-CTD region corresponding to the MBD of d-LDH into negative residues, to create repulsion towards the negative headgroups of lipids, membrane association is lost (Fig. 2 and Fig. 4a, NS4B-CTD tripleE), as well as the ability to form subgenomic replicon colonies (Fig. 5). Single substitution of each positive residue in the NS4B-CTD resulted in a mild loss of membrane targeting (Data not shown), though a complete loss of replicon colony formation (Fig. 5). It was previously shown that the integrity of NS4B is important for HCV replication; changes of only one amino acid can already influence replication [44,45]. Mutants in which we exchanged the complete membrane contacting helix of NS4B-CTD with the d-LDH membrane interface helix, retained membrane targeting (Fig. 2 and Fig. 4a, NS4B-CTD helix-swop) and selectable replicon colonies were obtained (Fig. 5). Nonetheless, in this NS4B-CTD helix-swop fewer replicon colonies were formed compared to wild type. Moreover the introduced sequence was unchanged in these colonies. In the NS4B-CTD helix-swop mutant eight amino acids are substituted and it gains in total one positively charged residue compared to NS4B-CTD, though this charge is distributed differently along the helix. Both the immunofluorescence assay and the colony formation assay illustrate that these mutations are allowed and indicate similar function of the two domains, NS4B-CTD and d-LDH MBD. Our experiments give an indication that the CTD of NS4B targets to membranes via electrostatic interactions of the positive residues in the NS4B-CTD with the negative phosphates of the phospholipids (Model in Fig. 6), moreover that this protein-membrane interaction is important for HCV RNA replication.

The NS4B protein is associated with membranes in various ways. Four to five transmembrane domains in the central region [20,23] and an amphipatic helix at the N-terminus of the protein [22] were described previously. In addition we now show that the CTD of NS4B is a membrane binding domain. This stresses the importance of protein-membrane interaction throughout the protein. For the NS4B-CTD we can envisage several possible functions. One possibility might be to position this domain of NS4B in a correct orientation. Recently, a membrane binding amphipatic helix in NS3, together with the transmembrane domain of NS4A, were suggested to properly position the NS3/4A protease on the membrane [11]. Positive residues in a MBD can also stabilize the orientation on the membrane surface [46]. In analogy, membrane contacts of NS4B-CTD might position the domain on the membrane surface or facing towards the cytosol.

NS4B is involved in the formation of membranous web structures [18]. Therefore, a function of the NS4B protein might be the induction of membrane curvature. The N-terminal amphipatic helix could act as a wedge inserted into one leaflet of the lipid bilayer leading to membrane curvature [47,48]. Also transmembrane domains can influence membrane curvature, depending on their conical shape [47]. The CTD of NS4B might induce or stabilize curvature by bracing the membrane like a scaffold [47,48].

An interesting question for both the N-terminal amphipatic helix and the CTD membrane binding domain of NS4B is whether these bind to the same membrane (cis) as the central transmembrane helices or that these can bind to other cellular membranes in close proximity (trans). In the latter situation it is conceivable that such a membrane-protein-membrane interaction would bring different membrane surfaces into close proximity, resulting in convoluted membranes [19].

Recently, the positively charged amino acids that we propose to interact with the lipid head groups, were also indicated in RNA-binding with an apparent preference for minus strand 3'NTR [25]. When we co-transfected NS4B-CTD together with an excess of minus strand 3'NTR RNA, no change in localization of the NS4B-CTD could be observed (data not shown). This indicates that membrane association is not affected by the suggested RNA binding characteristics of the domain in the presence of RNA.

Clearly, the HCV life cycle is achieved by the interchange between membranes, protein membrane anchors and proteins. The membranous web formation for replication, possibly lipid droplet associated membranes are involved in virus particle assembly [16,33]. The switch between active replication and assembly of infectious virus particles requires further levels of interactions between the membranous web and other associated membranes both in time and space [33,49,50]. The modular domain architecture and association to membranes of NS4B suggests various functions throughout these processes.

## Methods

### Antibodies

The following antibodies were used anti-PDI (Stressgen), anti-Myc (mouse) (Invitrogen), anti-Myc (rabbit) (Roche), anti-GAPDH (SantaCruz), anti-Transferrin receptor, clone H68.4 (Zymed Laboratories Inc), anti-COX-IV (Abcam), anti-Calnexin (BD) and anti-HA (Abcam).

### Cell culture and transfection

Human hepatoma cell line Huh7 was grown in Dulbecco's Modified Eagle's Medium supplemented with Non-essential amino acids, L-glutamate, Penicillin and Streptavadin. Cells were subcultured using Trypsin and transfected using Fugene6 (Roche) at a DNA/reagent ratio of 1/3, according to manufacturers' instructions.

### Plasmid Construction

To construct Myc-epitope-tagged expression plasmids, the sequence was amplified by PCR from pFK5.1Neo [51] or E.*coli *DNA using specific primers, see Table 1. The PCR products were digested with *Kpn*I and *Xba*I and ligated into pCDNA3.1mychisB (Invitrogen) similarly digested with *Kpn*I and *Xba*I. This resulted in the construction of expression vectors containing a 10-residue Myc-epitope-tag at its C-terminus. In order to construct HA-epitope-tagged NS4B expression constructs, the Myc-epitope sequence was *Xba*I – *Pme*I cut and replaced with an *Xba*I – *Pme*I fragment coding for the HA-epitope.

**Table 1:** Primers used to generate expression constructs

**NS4B FL**
Forward primer, GTGGGTACCATGTCACACCTCCCTTACATCGAACAG
Reverse primer, TAGTCTAGAGAGCCGGAGCATGGCGTGGAGCAGTC

**NS4B-CTD**

Forward primer, GTGGGTACCATGGCGATACTGCGTCGGCACGTGGGC
Reverse primer, as NS4B FL

**NS4B-deltaCTD**

Forward primer, as NS4B FL
Reverse primer, TAGTCTAGAGACCGACGCAGTATCGCTGCGCACACGAC

**NS4B-CTD sub-Cys**

Forward primer, as for NS4B-CTD
Reverse primer, AGATCTAGAGAGCCGGAGGATGGCGTGGAGGAGTCCTCGTTGATCCACTG

**d-LDH MBD**

Forward primer, GTGGGTACCATGAAATACGGCAAAGACACCTTCC
Reverse primer, TACTCTAGAGAATGCTCGTATTTATCGC

### In vitro transcription, electroporation and selection of selectable replicon cells

In vitro transcription, electroporation and selection of G418-resistant cell lines was done as described previously [52].

### Immunofluorescence microscopy

24 h post transfection cells were fixed with 3% paraformaldehyde (PFA) in PBS (154 mM NaCl, 1.4 mM Phosphate, pH 7.5). PFA was quenched using 50 mM NH4Cl in blockbuffer, which contained 5% fetal calf serum (FCS) in PBS. The cells were permeabilized with 0.1% TritonX-100 in blockbuffer and stained with primary antibodies diluted in blockbuffer for 1 h. Next the coverslips were washed with glycinebuffer, 10 mM glycine in PBS, and incubated with secondary antibody diluted in blockbuffer for 1 h. After washing with glycinebuffer, PBS and water, the coverslips were mounted with Prolong (Invitrogen) mounting medium. Fluorescence images were captured using a Zeiss Axioskop 2 fluorescence microscope equipped with the appropriate filter sets, a digital Axiocam HRc camera and Zeiss Axiovision 4.4 software. Images were optimized with Adobe Photoshop CS2.

### Floatation gradient

Transfected Huh7 cells were lysed after 24 h in buffer that contained 20 mM Tris pH 7, 1 mM MgCl_2_, 15 mM NaCl and 240 mM sucrose using a ball bearing homogenizer (Isobiotec, Heidelberg Germany). Whole cells and cell debris was spun down at 500 × g for 5 min and supernatant was collected. Cell extracts were mixed with sucrose to 80% w/v and overlaid with a linear sucrose gradient (80%–10% w/v sucrose, 50 mM Tris pH 7, 1 mM MgCl_2_, 15 mM NaCl). After centrifugation in a SW41 tube for 15 h at 100,000 × g (Beckmann ultracentrifuge), 500 μl fractions were collected from the top. The odd fractions were analyzed by western blotting, either directly or subsequent to concentration. 200 μl of each fraction was concentrated using 9 volumes of ethanol and incubated overnight at -20°C, followed by centrifugation at max in an Eppendorf 5417R for 1 h. The protein pellets were dissolved in 1× Laemmli.

### SDS-Page and western blotting

After separation on SDS-PAGE gels, proteins were transferred to PVDF membranes (HydrobondP, GE-Healthcare) using a Semi-Dry blot apparatus (Biorad). Membrane blocking and antibody incubations were performed using 0.5% Tween-20, 5% non-fat, dry milk (Campina) in PBS. Since all secondary antibodies were conjugated to horseradish peroxidase, the proteins were visualized using enzyme-catalyzed chemoluminescence (ECL+, GE-Healthcare) and Fuji Super RX medical X-ray film.

### Profile searches of sequence databases

COMPASS , database pfam21.0, 0 PSI-blast iterations, E-value threshold was set at 10. Profile comparer (PRC; ), database pfam22.0, E-value threshold was set at 10. HHpred , selected database pfamA_22.0, 0 PSI-blast iterations.

## Competing interests

The authors declare that they have no competing interests.

## Authors' contributions

JMPL performed all biochemical experiments, participated in the design of the study and wrote the manuscript. BWB was responsible for the profile searches and participated in drafting the manuscript. RB constructed mutants and critically read the manuscript. WJMS was involved in revising the manuscript critically and participated in supervision of the study. HCVL drafted the manuscript, supervised and designed the study.

## Supplementary Material

Additional file 1 **Multiple sequence alignment of NS4B carboxy terminal domain and Lact-deh-memb**. Top panel contains a multiple sequence alignment of Lact-deh-memb (PF09330). A multiple sequence alignment of the NS4B-CTD, which includes all genotypes of HCV, Hepatitis GB virus A, B and C is shown in the bottom panel. Click here for file
