# Supplementary material for: Hepatitis C virus NS4B carboxy terminal domain is a membrane binding domain
Source: Virol J. 2009 May 25;6:62. doi: 10.1186/1743-422X-6-62 (PMC2698844; doi:10.1186/1743-422X-6-62)
Supplement: Additional file 1 — Multiple sequence alignment of NS4B carboxy terminal domain and Lact-deh-memb. Top panel contains a multiple sequence alignment of Lact-deh-memb (PF09330). A multiple sequence alignment of the NS4B-CTD, which includes all genotypes of HCV, Hepatitis GB virus A, B and C is shown in the bottom panel. [file 1743-422X-6-62-S1.pdf]

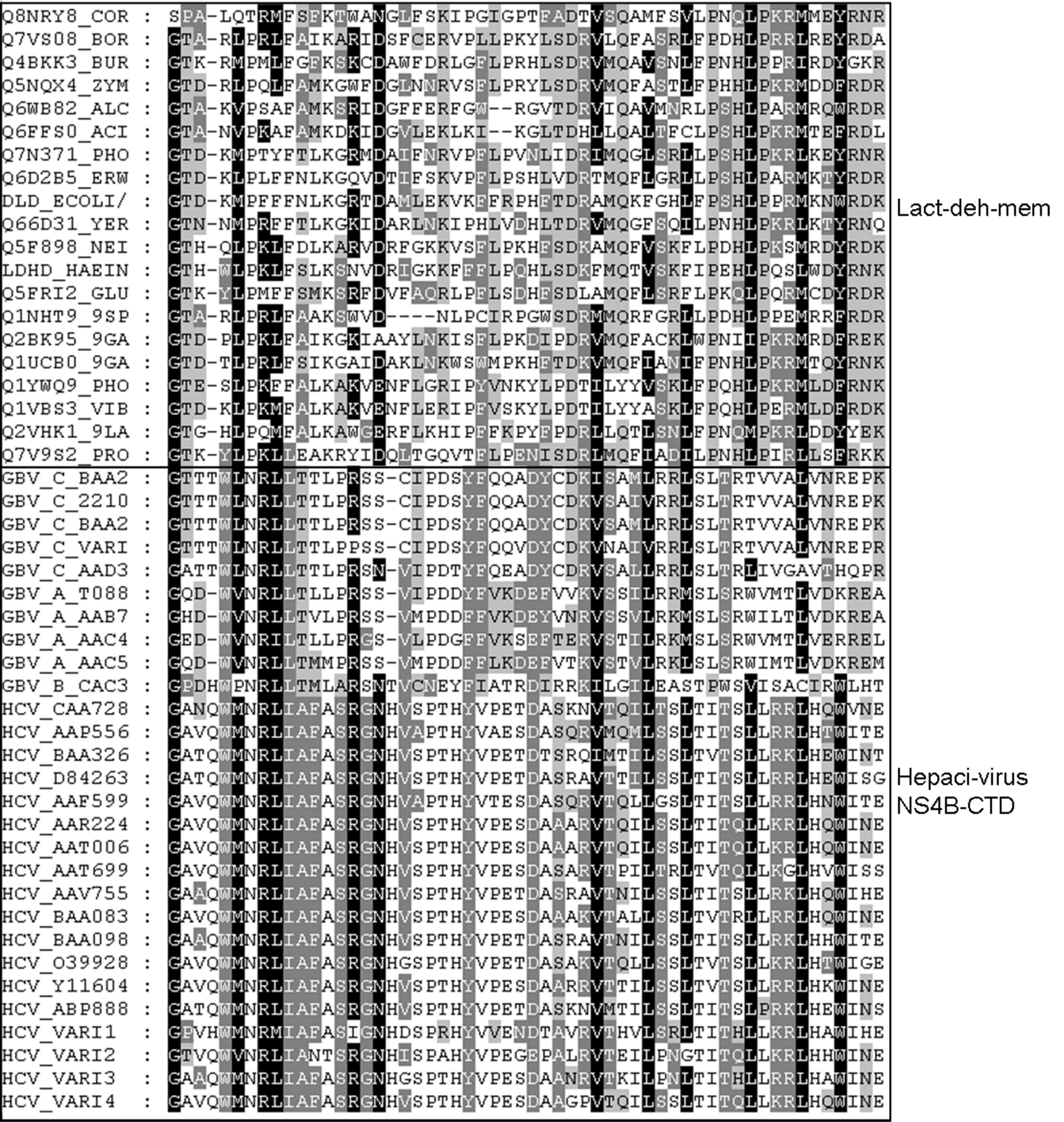
Q8NRY8_COR : SPA-LQTRMFSFKTWANGLFSKIPGIGPTFADTVSQAMFSVLPNQLPKRMMEYRNR
Q7VS08_BOR : GTA-RLPRLFAIKARIDSFCERVPLLPKYLSDRVLQFASRLFPDHLPRRLREYRDA
Q4BKK3_BUR : GTK-RMPMLFGFKSKCDAWFDRLGFLPRHLSDRVMQAVSNLFPNHLPPRIRDYGKR
Q5NQX4_ZYM : GTD-RLPQLFAMKGWFDGLNNRVSFLPRYLSDRVMQFASTLFPHHLPKRMDDFRDR
Q6WB82_ALC : GTA-KVPSAFAMKSRIDGFFERFGW--RGVTDRVIQAVMNRLPSHLPARMRQWRDR
Q6FFS0_ACI : GTA-NVPKAFAMKDKIDGVLEKLKI--KGLTDHLLQALTFCLPSHLPKRMTEFRDL
Q7N371_PHO : GTD-KMPTYFTLKGRMDAIFNRVPFLPVNLIDRIMQGLSRLLPSHLPKRLKEYRNR
Q6D2B5_ERW : GTD-KLPLFFNLKGQVDTIFSKVPFLPSHLVDRTMQFLGRLLPSHLPARMKTYRDR
DLD_ECOLI/ : GTD-KMPFFFNLKGRTDAMLEKVKEFRPHETDRAMQKFGHLFPSHLPPRMKNWRDK
Q66D31_YER : GTN-NMPRFFTLKGKIDARLNKIPHLVDHLTDRVMQGFSQILPNHLPKRLKTYRNQ
Q5F898_NEI : GTH-QLPKLFDLKARVDRFGKKVSFLPKHFSDKAMQFVSKFLPDHLPKSMRDYRDK
LDHD_HAEIN : GTH-WLPKLFSLKSNVDRIGKKFFFLPQHLSDKFMQTVSKFIPEHLPQSLWDYRNK
Q5FRI2_GLU : GTK-YLPMFFSMKSRFDVFAQRLPFLSDHFSDLAMQFLSRFLPKQLPQRMCDYRDR
Q1NHT9_9SP : GTA-RLPRLFAAKSWVD----NLPCIRPGWSDRMMQRFGRLLPDHLPPEMRRFRDR
Q2BK95_9GA : GTD-PLPKLFAIKGKIAAYLNKISFLPKDIPDRVMQFACKLWPNIIPKRMRDFREK
Q1UCB0_9GA : GTD-TLPRLFSIKGAIDAKLNKWSWMPKHFTDKVMQFIANIFPNHLPKRMTQYRNK
Q1YWQ9_PHO : GTE-SLPKFFALKAKVENFLGRIPYVNKYLPDTILYYVSKLFPQHLPKRMLDFRNK
Q1VBS3_VIB : GTD-KLPKMFALKAKVENFLERIPFVSKYLPDTILYYASKLFPQHLPERMLDFRDK
Q2VHK1_9LA : GTG-HLPQMFALKAWGERFLKHIPFFKPYFPDRLLQTLSNLFPNQMPKRLDDYYEK
Q7V9S2_PRO : GTK-YLPKLLEAKRYIDQLTGQVTFLPENISDRLMQFIADILPNHLPIRLLSFRKK
Lact-deh-mem
GBV_C_BAA2 : GTTTWLNRLLTTLPRSS-CIPDSYFQQADYCDKISAMLRRLSLTRTVVALVNREPK
GBV_C_2210 : GTTTWLNRLLTTLPRSS-CIPDSYFQQADYCDKVSAIVRRLSLTRTVVALVNREPK
GBV_C_BAA2 : GTTTWLNRLLTTLPRSS-CIPDSYFQQADYCDKVSAMLRRLSLTRTVVALVNREPK
GBV_C_VARI : GTTTWLNRLLTTLPPSS-CIPDSYFQQVDYCDKVNAIVRRLSLTRTVVALVNREPR
GBV_C_AAD3 : GATTWLNRLLTTLPRSN-VIPDTYFQEADYCDRVSALLRRLSLTRLIVGAVTHQPR
GBV_A_T088 : GQD-WVNRLLTLLPRSS-VIPDDYFVKDEFVVKVSSILRRMSLSRWVMTLVDKREA
GBV_A_AAB7 : GHD-WVNRLLTVLPRSS-VMPDDFFVKDEYVNRVSSVLRKMSLSRWILTLVDKREA
GBV_A_AAC4 : GED-WVNRILTLLPRGS-VLPDGFFVKSEFTERVSTILRKMSLSRWVMTLVERREL
GBV_A_AAC5 : GQD-WVNRLLTMMPRSS-VMPDDFFLKDEFVTKVSTVLRKLSLSRWIMTLVDKREM
GBV_B_CAC3 : GPDHWPNRLLTMLARSNTVCNEYFIATRDIRRKILGILEASTPWSVISACIRWLHT
HCV_CAA728 : GANQWMNRLIAFASRGNHVSPTHYVPETDASKNVTQILTSLTITSLLRRLHQWVNE
HCV_AAP556 : GAVQWMNRLIAFASRGNHVAPTHYVAESDASQRVMQMLSSLTITSLLRRLHTWITE
HCV_BAA326 : GATQWMNRLIAFASRGNHVSPTHYVPETDTSRQIMTILSSLTVTSLLRKLHEWINT
HCV_D84263 : GATQWMNRLIAFASRGNHVSPTHYVPETDASRAVTTILSSLTITSLLRRLHEWISG
HCV_AAF599 : GAVQWMNRLIAFASRGNHVAPTHYVTESDASQRVTQLLGSLTITSLLRRLHNWITE
HCV_AAR224 : GAVQWMNRLIAFASRGNHVSPTHYVPESDAAARVTQILSSLTITQLLKRLHQWINE
HCV_AAT006 : GAVQWMNRLIAFASRGNHVSPTHYVPESDAAARVTQILSSLTITQLLKRLHQWINE
HCV_AAT699 : GAVQWMNRLIAFASRGNHVSPTHYVPESDASARVTPILTRLTVTQLLKGLHVWISS
HCV_AAV755 : GAAQWMNRLIAFASRGNHVSPTHYVPETDASRAVTNILSSLTITSLLRKLHQWIHE
HCV_BAA083 : GAVQWMNRLIAFASRGNHVSPTHYVPESDAAAKVTALLSSLTVTRLLRRLHQWINE
HCV_BAA098 : GAAQWMNRLIAFASRGNHVSPTHYVPETDASRAVTNILSSLTITSLLRKLHHWITE
HCV_O39928 : GAVQWMNRLIAFASRGNHGSPTHYVPETDASAKVTQLLSSLTVTSLLKRLHTWIGE
HCV_Y11604 : GAVQWMNRLIAFASRGNHVSPTHYVPESDAARRVTTILSSLTVTSLLRRLHKWINE
HCV_ABP888 : GATQWMNRLIAFASRGNHVSPTHYVPETDASKNVMTILSSLTITSLPRKLHEWINS
HCV_VARI1 : GPVHWMNRMIAFASIGNHDSPRHYVVENDTAVRVTHVLSRLTITHLLKRLHAWIHE
HCV_VARI2 : GTVQWVNRLIANTSRGNHISPAHYVPEGEPALRVTEILPNGTITQLLKRLHHWINE
HCV_VARI3 : GAAQWMNRLIAFASRGNHGSPTHYVPESDAANRVTKILPNLTITHLLRRLHAWINE
HCV_VARI4 : GAVQWMNRLIAFASRGNHVSPTHYVPESDAAGPVTQILSSLTITQLLKRLHQWINE
Hepaci-virus
NS4B-CTD
